# Targeted Haplotype Comparisons between South African Wheat Cultivars Appear Predictive of Pre-harvest Sprouting Tolerance

**DOI:** 10.3389/fpls.2018.00063

**Published:** 2018-02-01

**Authors:** Scott L. Sydenham, Annelie Barnard

**Affiliations:** Agricultural Research Council-Small Grain, Bethlehem, South Africa

**Keywords:** haplotype, pre-harvest sprouting, *TaPHS1*, *Phs1-A1*, QTL, SSRs, wheat

## Abstract

Pre-harvest sprouting (PHS) has been a serious production constraint for over two decades, especially in the summer rainfall wheat production regions of South Africa. It is a complex genetic trait controlled by multiple genes, which are significantly influenced by environmental conditions. This complicates the accurate prediction of a cultivar's stability in terms of PHS tolerance. A number of reports have documented the presence of major QTL on chromosomes 3A and 4A of modern bread wheat cultivars, which confer PHS tolerance. In this study, the SSR marker haplotype combination of chromosomes 3A and 4A of former and current South African cultivars were compared with the aim to select for improved PHS tolerance levels in future cultivars. A total of 101 wheat cultivars, including a susceptible cultivar and five international tolerant sources, were used in this study. These cultivars and donors were evaluated for their PHS tolerance by making use of a rain simulator. In addition, five seeds of each entry were planted out into seedling trays and leaf material harvested for DNA isolation. A modified CTAB extraction method was used before progressing to downstream PCR applications. Eight SSR markers targeted from the well-characterized 3A and 4A QTL regions associated with PHS tolerance, were used to conduct targeted haplotype analysis. Additionally, recently published KASP SNP markers, which identify the casual SNP mutations within the *TaPHS1* gene, were used to genotype the germplasm. The haplotype marker data and phenotypic PHS data were compared across all cultivars and different production regions. A relative change in observed phenotypic variation percentage was obtained per marker allele and across marker haplotype combinations when compared to the PHS susceptible cultivar, Tugela-DN. Clear favorable haplotypes, contributing 40–60% of the variation for PHS tolerance, were identified for QTL 3A and 4A. Initial analyses show haplotype data appear to be predictive of PHS tolerance status and germplasm can now be selected to improve PHS tolerance. These haplotype data are the first of its kind for PHS genotyping in South Africa. In future, this can be used as a tool to predict the possible PHS tolerance range of a new cultivar.

## Introduction

Pre-harvest sprouting (PHS) is a common phenomenon in the wheat (*Triticum aestivum* L.) producing areas of South Africa and has been well-documented over the past two decades (Barnard et al., 1997; Barnard, 2001; Barnard and Bona, 2004; Barnard and Smith, 2009). It has been a serious production constraint especially in the summer rainfall regions where rain occurs frequently just prior to or during harvest time. It is well-documented that PHS negatively affects the grain quality and ultimately flour quality. As a result, the price that farmers can get for their crop at harvest is severely affected (Barnard, 2001; Liu et al., 2008).

Research has shown that extensive genotypic variation exists for PHS in South African cultivars, indicating that progress in the development of cultivars with improved sprouting tolerance is feasible (Barnard et al., 1997, 2005; Barnard, 2001). The PHS tolerance levels in South African wheat cultivars has improved significantly over the years as a result of successful breeding (Smit et al., 2010). These winter wheat cultivars can be categorized into three major groups, namely cultivars that are highly tolerant to PHS, cultivars that are highly susceptible to PHS and a third moderate group that includes cultivars that are strongly influenced by the environment (Barnard and Smith, 2009). According to Biddulph et al. (2005) environment, and specifically moisture stress, can have a large effect on dormancy expression. Drought conditions combined with high temperatures during grain filling, tend to increase dormancy in wheat (Mares and Mrva, 2014).

PHS is a complex trait controlled by multiple genes or QTL (Bailey et al., 1999; Mares et al., 2005; Yang et al., 2007) where trait expression is significantly influenced by environmental conditions (Trethowan et al., 1996; Johansson, 2002). This complicates the accurate prediction of the stability of a cultivar in terms of PHS tolerance.

In the past decade, a number of QTL for PHS tolerance have been identified and mapped across all 21 wheat chromosomes in a number of wheat cultivars from different parts of the world (Mori et al., 2005; Ogbonnaya et al., 2007; Chen et al., 2008; Mohan et al., 2009; Jaiswal et al., 2012; Singh et al., 2012; Graybosch et al., 2013). These QTL analyses in wheat led to the identification of markers linked closely with desirable alleles of different QTL (Mares et al., 2005; Chen et al., 2008; Liu et al., 2008; Fofana et al., 2009; Kulwal et al., 2010, 2012). The chromosomes containing the most common and stable major QTL for PHS tolerance are 3A (Kulwal et al., 2005) and 4A (Mares et al., 2005; Mori et al., 2005; Ogbonnaya et al., 2007; Chen et al., 2008; Imtiaz et al., 2008; Zhang et al., 2008). A number of robust reliable simple-sequence repeat (SSR) markers have been associated to a number of these specific QTL for PHS tolerance in specific cultivar backgrounds. However, the characterization and validation of the true phenotypic effects of these QTL individually or in combination in diverse germplasm remains a challenge due to the genetic complexity of the PHS tolerance trait.

The major QTL on chromosome 4A was identified and mapped in 2000 (Flintham, 2000), which is now referred to as the *Phs1*-*A1* locus (Shorinola et al., 2016). Recently, the *Phs1-A1* region was fine mapped and new tightly molecular markers with MAS potential were identified. However, the causal gene underpinning the *Ph1-A1* locus is still unclear (Barrero et al., 2015; Shorinola et al., 2016). In 2008, a major QTL on chromosome 3A, named *Qphs.pseru-3AS*, was characterized and mapped from the white wheat cultivar Rio Blanco (Liu et al., 2008). In recent years, some important candidate genes which control PHS tolerance at these (3A and 4A) loci and others have been identified (Liu et al., 2013; Cabral et al., 2014; Barrero et al., 2015; Shorinola et al., 2016; Zhou et al., 2017). Importantly, the TaPHS1 gene, which forms an integral part of the major QTL on chromosome 3A (*Qphs.pseru-3AS*), which confers PHS tolerance, was cloned and characterized further. Two important, functional SNP mutations within the third and fourth exons of the *TaPHS1* gene-coding region, were identified. Both SNP mutations occurred together in all PHS susceptible cultivars covering a set of diverse genetic backgrounds and are considered critical for future PHS tolerant cultivar development (Liu et al., 2013).

The aim of this study was to characterize a collection of South African wheat cultivars for their known PHS tolerance QTL on chromosomes 3A and 4A and to compare marker haplotype combinations observed with the original PHS cultivar scoring averages. In this study, we aim to validate whether these markers could be used during MAS to select for better PHS tolerant cultivars and to determine if it would be possible to predict a cultivar's potential PHS tolerant class based solely on marker haplotypes.

## Materials and methods

### Wheat cultivars and trials

A total of 96 red wheat cultivars (Table 1) were included in this study and evaluated for their PHS tolerance or susceptibility over a 20 year-period and across six environments per year. These cultivars from three different seed companies (ARC-Small Grain, Pannar and Sensako), were commonly grown under dryland conditions in the summer rainfall dryland area, as well as under irrigation conditions in the central wheat producing areas of South Africa. Tugela-DN was used as a susceptible check, while Elands was included as a tolerant check (Barnard et al., 2005). The cultivars were planted according to a randomized complete block design (RCBD) with four replicates and accessed annually for the period that they were commercially available. Five sources of PHS tolerance namely AC Domain (Fofana et al., 2009) RL4137 (DePauw et al., 2009), Renan (Groos et al., 2002), Transvaal (Morris and DeMacon, 1994) and Rio Blanco (Liu et al., 2008), were also evaluated for their PHS characteristics over the last 3 years.

**Table 1:** The PHS phenotypic data of 96 wheat cultivars commonly grown in South Africa over multiple years and seasons.

	**Dryland cultivars**				**Irrigation cultivars**		
	**Year released**	**# years evaluated**	**Mean PHS score ± SD**		**Year released**	**# years evaluated**	**Mean PHS score ± SD**
Betta	1969	4	1.5 ± 0.33	Adam Tas	1989	3	5.8 ± 0.50
Betta-DN	1993	13	2.1 ± 0.92	Baviaans	2000	11	2.9 ± 0.43
Caledon	1996	15	2.7 ± 0.69	Biedou	2001	1	2.9
Elands	1998	17	2.0 ± 0.71	Buffels	2007	6	2.5 ± 0.26
Flamink	1979	1	6.8	Chokka	1989	2	4.6 ± 0.77
Gariep	1994	18	3.5 ± 0.48	CRN 826	2002	10	4.4 ± 0.59
Hugenoot	1989	9	4.8 ± 1.49	Dias	1988	1	5.4
Karee	1982	8	2.1 ± 0.69	Duzi	2004	11	3.7 ± 0.38
Komati	2002	6	2.0 ± 0.41	Gamtoos	1985	4	3.9 ± 0.99
Koonap	2010	4	3.9 ± 0.53	Inia	1970	9	4.1 ± 0.55
Letaba	1987	3	3.2 ± 1.06	Kariega	1993	17	2.5 ± 0.70
Limpopo	1994	11	3.1 ± 0.94	Krokodil	2004	11	4.1 ± 0.55
Matlabas	2004	11	2.7 ± 0.56	Marico	1993	12	3.1 ± 1.14
Molen	1986	5	5.4 ± 0.97	Nantes	1990	3	3.9 ± 0.89
Molopo	1988	3	3.2 ± 1.94	Olifants	2001	11	4.9 ± 0.93
Oom Charl	1987	3	1.9 ± 0.81	Palmiet	1985	6	4.4 ± 1.12
PAN 3111	2012	2	4.4 ± 0.57	PAN 3400	2011	3	4.2 ± 1.05
PAN 3118	2001	12	3.8 ± 0.81	PAN 3434	2004	7	3.5 ± 0.55
PAN 3120	2002	11	2.6 ± 0.56	PAN 3471	2008	7	4.9 ± 0.69
PAN 3122	2002	2	4.5 ± 0.42	PAN 3478	2008	6	3.3 ± 0.30
PAN 3144	2005	6	2.7 ± 0.48	PAN 3489	2011	3	4.8 ± 0.68
PAN 3161	2007	7	4.5 ± 0.58	PAN 3497	2011	3	3.4 ± 0.35
PAN 3191	1999	6	3.8 ± 1.44	PAN 3515	2013	1	3.2
PAN 3195	2011	3	5.4 ± 0.46	PAN 3623	2013	1	2.5
PAN 3198	2012	2	4.5 ± 0.71	Sabie	2010	6	2.8 ± 0.56
PAN 3355	2006	6	3.0 ± 0.49	SST 38	1993	6	2.9 ± 0.61
PAN 3364	1996	7	2.3 ± 0.82	SST 806	2000	13	4.8 ± 0.55
PAN 3368	2007	7	2.4 ± 0.54	SST 822	1992	18	3.8 ± 0.91
PAN 3377	1997	9	3.3 ± 1.03	SST 825	1992	9	5.4 ± 0.49
PAN 3379	2007	7	3.6 ± 0.33	SST 835	2003	10	4.6 ± 0.61
Scheepers 69	1969	2	2.0 ± 0.28	SST 843	2008	7	4.5 ± 0.60
Senqu	2010	4	2.7 ± 0.15	SST 866	2011	5	4.0 ± 0.58
SST 124	1987	10	3.7 ± 1.65	SST 867	2009	5	2.5 ± 0.44
SST 316	2013	3	3.8 ± 0.67	SST 875	2012	5	4.3 ± 0.72
SST 317	2013	3	2.8 ± 0.06	SST 876	1997	14	5.6 ± 0.62
SST 322	2002	4	2.4 ± 0.54	SST 877	2010	5	2.3 ± 0.28
SST 347	2004	7	2.7 ± 0.60	SST 884	2013	4	4.7 ± 0.91
SST 356	2005	8	3.5 ± 0.36	SST 895	2014	4	3.2 ± 0.71
SST 374	2011	2	3.0 ± 0.85	SST 896	2014	1	5.0
SST 387	2012	5	3.8 ± 0.54	SST 16	1988	3	5.7 ± 1.25
SST 398	2010	4	2.7 ± 1.04	SST 33	1988	3	4.5 ± 1.54
SST 399	1999	7	2.8 ± 0.44	SST 44	1988	1	6.1
SST 935	2003	2	4.7 ± 0.07	SST 66	1988	4	6.0 ± 0.56
SST 936	1994	4	3.5 ± 0.39	SST 86	1988	2	3.3 ± 0.25
SST 946	2004	1	3.6	Steenbras	1999	10	4.9 ± 0.57
Tugela	1986	5	7.2 ± 0.16	T4	1965	6	2.3 ± 0.80
Tugela-DN	1992	25	6.4 ± 0.89	Tamboti	2011	3	3.4 ± 0.32
				Timbavati	2011	3	3.3 ± 0.85
				Umlazi	2010	3	3.3 ± 0.23

### Assessment of PHS

**Figure 1:**
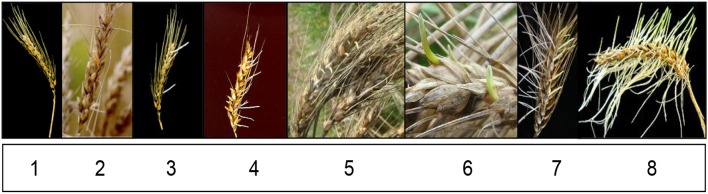
Evaluation scale to determine the PHS tolerant or susceptibility of cultivars.

During anthesis 48 ears per cultivar were labeled to ensure that all the ears were at the same physiological stage. These ears were hand-harvested at physiological maturity and air dried at room temperature for a week. The ears were then subjected to simulated rainfall for 72 h in a rain simulator at 15°C/25°C day/night temperature with 98% humidity as described by Barnard et al. (1997). According to this technique, individual ears were evaluated on a scale from 1 to 8, where 1 represents total tolerance to PHS and 8 represents total susceptibility (Figure 1). The PHS phenotypic data collected, were averaged per cultivar.

### DNA isolation

Five seeds of each entry were planted out into seedling trays. Seven days post seedling emergence, fresh leaf material was harvested for DNA isolation. The leaf tissue was homogenized finely within 750 μl of extraction buffer for 1 min at 30 r/s with the Qiagen TissueLyser II. Genomic DNA was isolated according to a modified cetyltrimethylammonium bromide (CTAB) DNA extraction protocol by Saghai-Maroof et al. (1994) and treated with 2 μl RNase A enzyme (Inqaba Biotechnology). The quality, purity and concentration of each DNA sample was determined at 260/280 nm with a Nanodrop 2000 Spectrophotometer (Thermo Scientific Pty Ltd, USA). The DNA samples were then diluted with 1x TE (Tris-EDTA) buffer to 50 ng/μl before progressing to downstream PCR applications.

### Markers used

All SSR marker primer pairs were synthesized by Integrated DNA Technologies (www.IDTDNA.com) and ordered through Whitehead Scientific PTY (Ltd) (www.whitesci.co.za). Initially, 31 different SSR marker primer sequences and relevant PCR conditions were obtained either from Röder et al. (1998) and/or the grain genes 2.0 website (https://wheat.pw.usda.gov/GG2/). These 31 SSR markers were screened on seven local cultivars and the five international sources to identify informative polymorphic markers. Table 2 lists these SSR markers, as well as the targeted PHST QTL per chromosome, and describes whether these markers were informative or not. From the initial screening, four polymorphic SSR markers were identified for potential targeted haplotype combination analysis. These markers, namely *Barc57* and *Barc12* (3A QTL) and *DuPw004* and *Wmc650* (4A QTL) were targeted from the well-characterized 3A and 4A QTL regions associated with PHS tolerance. The 96 cultivars, as well as the five international PHS tolerant donors were genotyped with the four SSR markers.

**Table 2:** List of the SSR markers that were used during the initial screening phase of this study, together with their targeted chromosomes.

**SSR Marker**	**Target QTL**	**Status**	**Comments**
*Wmc650*	Major PHS 4A QTL	Polymorphic	Informative
*Barc170*		Polymorphic	Mostly informative
*DuPw004*		Polymorphic	Informative
*Gwm397*		Polymorphic	Mostly informative
*Xgwm269*
*Wmc48*	4AL	Polymorphic	Informative certain tolerant material
*Wmc491*	4AL	Polymorphic	Not reliable
*Wmc680*	4AL	Polymorphic	Mostly informative
*Wmc707*	4AL	Polymorphic	Mostly informative
*gwm494*	4AL	Monomorphic	Not Informative
*Barc57*	Major PHS 3A QTL	Polymorphic	Informative
*Barc12*		Polymorphic	Informative
*Barc321*		Polymorphic	Not informative
*Gwm403*	3AL	Polymorphic	Mostly informative
*Wmc428*	3AL	Monomorphic	Not Informative
*Wmc96*	3AL	Monomorphic	Not Informative
*gdm99*	3AL	Monomorphic	Not Informative
*Wwmc664*	3AS	Unreliable	Not Informative
*Gwm32*	3AS	Monomorphic	Not Informative
*Gwm4*	3AS	Monomorphic	Not Informative
*Gwm5*	2D/3AS	Polymorphic	Not informative
*Wmc492*	3DS	Polymorphic	Not Informative
*Wmc656*	3DL	Unreliable	Not Informative
*Gwm456*	3DL	Polymorphic	Informative on certain tolerant material
*Gwm3*	3D	Polymorphic	Not Informative
*Wmc349*	4BS	Monomorphic	Not Informative
*Wmc413*	4BS	Monomorphic	Not Informative
*Xgwm6*	4BS	Did not work	Not Informative
*Wmc657*	4BL	Polymorphic	Informative on certain tolerant material
*gwm63*	7AL	Unreliable	Not Informative
*Gwm37*	7DL	Unreliable	Not Informative

### Simple sequence repeat analysis

Extracted genomic DNA, totalling a 200 ng (4 μl) concentration was used as template DNA per sample in a 20 μl final volume PCR reaction. Reaction conditions recommended for the KAPA 2X Ready Mix PCR Kit (KAPA Biosystems, Cape Town, South Africa, www.kapabiosystems.com were applied. Each PCR reaction consisted of 10 μl (1x) KAPATaq 2X Ready Mix, 0.5 μl (10 μM) per SSR primer and the remaining volume (5.0 μl) of DNAse Free water. The PCR reactions were performed in a MyCyclerTM Thermal Cycler (www.bio-rad.com) with the following cycling conditions: 3 min at 95°C, 40 cycles of 30 s at 95°C, 30 s at Tm°C, 30 s at 72°C and a final extension step of 5 min at 72°C. After amplification each specific SSR marker PCR amplicons were separated on a 3.0–3.5% (w/v) Certified Low Range Ultra Agarose high-resolution gel (Bio-Rad Laboratories, Inc. www.bio-rad.com), made up in 1x TBE with 1x GRGreen Nucleic Acid gel stain solution (Inqaba Biotechnology, www.labsupplymall.com) and run at 100–125 V for 1–4 h. SSR product sizes were determined according to 100 bp and/or 20 bp (Lonza SimplyLoad®, Lonza Rockland Inc. USA) DNA ladders. A digital gel picture under UV light exposure was taken with the Bio-Rad Molecular Imager Gel Doc™ XR Instrument. Observed SSR marker alleles were sized, recorded and analyzed per cultivar both visually and with image Lab™ gel analysis software.

### KASPR marker genotyping

Two KASP assays, namely *TaPHS1-646* (TaPHS1-SNP1 marker) and *TaPHS1-666* (TaPHS1-SNP2 marker), designed during the study of Liu et al. (2013), are considered the functional SNP mutations in and around the *TaPHS1* gene region on chromosome 3A. These two KASP assays were screened on the 64 cultivars that were assigned haplotypes based on SSR markers, and the five international tolerant sources. The primer sequences of each assay and PCR condition were obtained from the MAS Wheat Website (http://maswheat.ucdavis.edu/protocols/TaPHS1/index.htm). The PCR reactions and fluorescence detection were performed in an Agilent Technologies Mx3500P Real-time Thermal Cycler as recommended by LGC (http://www.kbioscience.co.uk).

The specific SNP allele for each KASP marker was recorded per cultivar. When one of the unfavorable alleles for either SNP marker was present, a cultivar was predicted as susceptible. When the allele that was present was favorable, but the other allele was missing, a cultivar was treated as unknown. When both alleles were missing, a cultivar was also treated as unknown.

### Data analyses

Four SSR markers, namely *Barc57* and *Barc12* (3A QTL) and *DuPw004* and *Wmc650* (4A QTL), were used in the final haplotype analyses of the 3A and 4A QTL. Additive allele identification was performed based on average PHS data for a particular marker haplotype combination on the comparison of mean PHS scores of the susceptible check, Tugela-DN. Mean PHS scores per SSR allele were used to calculate the percentage change in observed phenotypic variation in PHS tolerance from the susceptible check. This was done regardless of genetic background to attempt to reduce the effect that different genetic backgrounds might have on observed PHS tolerance levels. Tugela-DN was used as the susceptible check as a point of reference in the observed phenotypic variation analysis. The average PHS score per marker allele containing multiple genotypes was deducted from the average PHS score of the susceptible cultivar (Tugela-DN) and then divided by the Tugela-DN average to get an observed phenotypic variation percentage. The alleles were then classed as tolerant, moderate or susceptible based on these PHS averages.

*Example:* Marker 1, Allele 1 = 6.4 (Tugela-DN)–2.9 (Marker 1/Allele 1) = 3.5/6.4 = 54.7% relative observed phenotypic variation (OPV).

## Results

### PHS characterization

The 96 cultivars used in this study are listed alphabetically in Table 1. These cultivars released from the late 1960's onwards were evaluated over a period of 25 years. Since new cultivars were released each year and older cultivars withdrawn from the market, it was difficult to evaluate these cultivars for similar periods of time. The number of years that the cultivars were evaluated for their PHS tolerance is therefore also shown in Table 1. Tugela-DN was released as a commercial cultivar in 1992 and has been the susceptible check since, with an average PHS value of 6.4. Elands, released in 1998, has an average PHS value of 2.0 and has been the tolerant check for the last 20 years.

The five international sources, namely AC Domain, RL4137, Rio Blanco, Transvaal and Renan, all had low PHS scores, namely 1.1, 1.2, 1.2, 1.6, and 1.1, respectively. This indicates excellent PHS tolerance.

The PHS tolerance levels of the cultivars in the study varied from excellent (scores lower than 3.0) to moderate (scores between 3.0 and 4.5) to highly susceptible (scores higher than 4.5). The cultivars adapted to dryland conditions were more tolerant to PHS with 43% of the entries having excellent tolerance to PHS, compared to the 20% of excellent tolerance in irrigation cultivars. The number of cultivars with moderate tolerance was similar in both groupings (43 and 47%, respectively, for dryland and irrigation cultivars).

**Figure 2:**
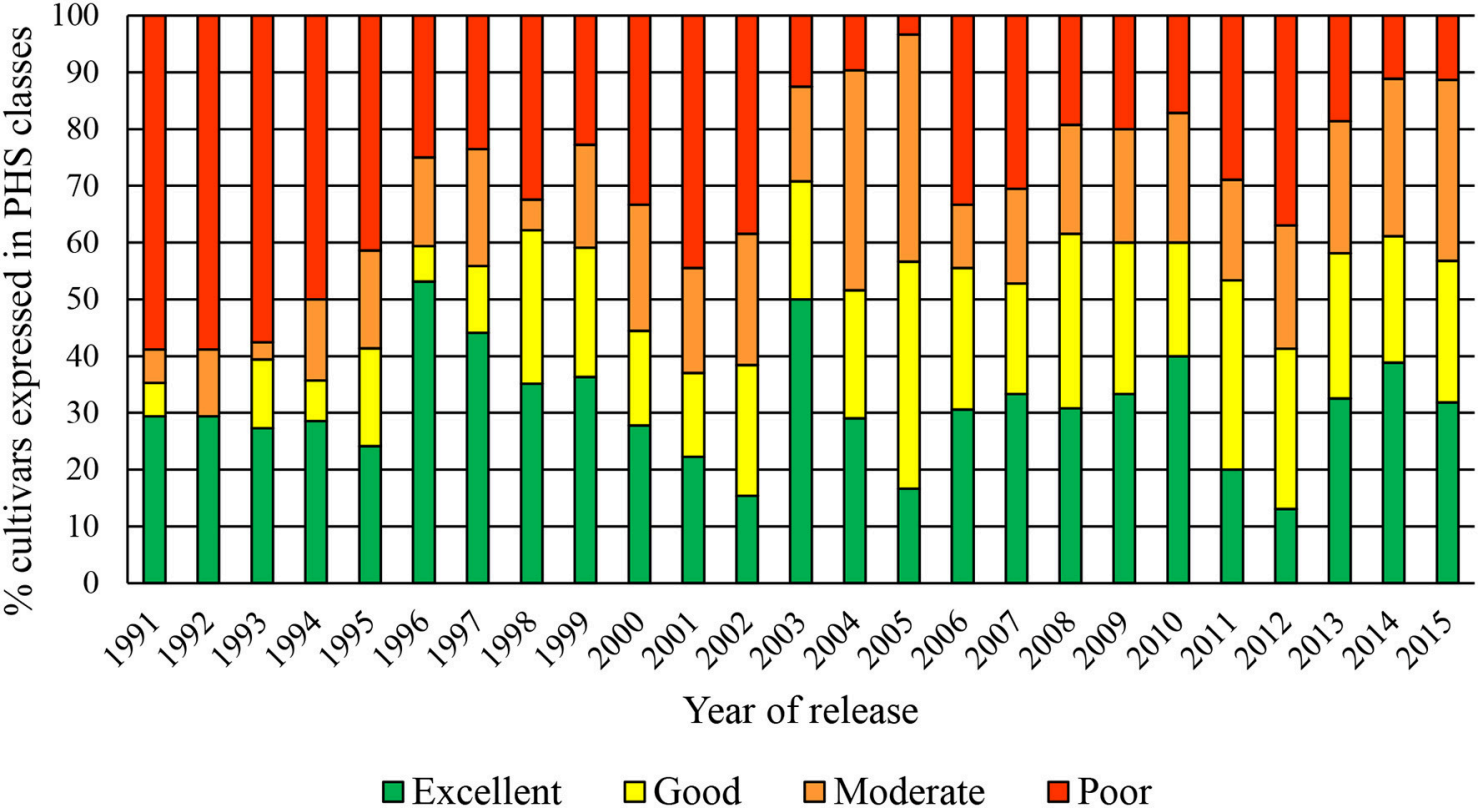
Evaluation scale to determine the PHS tolerant or susceptibility of cultivars.

Figure 2 shows the cumulative PHS data over the past 25 years. From these data in it is clear that the older cultivars (released in the previous millennium) had poorer tolerance than cultivars released after 2002. This was especially true for the dryland cultivars. The higher number of susceptible cultivars released in 2006, 2007, 2011, and 2012, were mainly irrigation cultivars.

### Favorable marker allele identification

Alleles were considered favorable for PHS tolerance when representative cultivars had PHS average scores of 3.0 or lower. An allele was classified as moderate if the average PHS scores ranged from 3.1 to 4.4. Finally, a marker allele was considered unfavorable for PHS tolerance if the average PHS scores of the representative cultivars were 4.5 or higher.

In Table 3 the single marker alleles for markers flanking the 3A QTL, *Barc57* and *Barc12*, and their relative observed phenotypic variation percentage are shown.

**Table 3:** Analysis of markers *Barc57* and *Barc12* that flank the 3A QTL to identify favorable alleles for PHS tolerance and the relative observed phenotypic variation (%) based on rain simulator screening of 96 wheat cultivars.

***Barc57***				***Barc12***			
**Allele**	Mean PHS score^*^	OPV%^**^	**Range**	**Allele**	**Mean PHS score**	**OPV%**	**Range**
220	3.2	50.0	1.5–7.2	220	3.3	48.4	1.5–4.9
210	3.6	43.8	2.4–4.9	200	3.6	43.8	2.0–5.4
210/240	4.1	35.9	2.0–5.4	160	3.8	40.6	1.9–6.1
220/240	4.1	35.9	2.3–6.4	180	3.9	39.1	2.8–5.0
				210	3.9	39.1	2.6–4.6
				240	4.8	25.0	2.3–6.4

* *PHS, Pre-harvest sprouting*.

** *OPV, Observed phenotypic variation*.

#### Barc57

SSR marker *Barc57* amplified five different alleles across the cultivars studied (Table 3). Allele *Barc57*^220/240^ contributed 53.1% to the observed phenotypic variation (OPV), with an average PHS score of 3.0 and appears to be more favorable for PHS tolerance than alleles *Barc57*^210/240^ and *Barc57*^210^. The *Barc57*^220^ allele, with a 40.6% contribution to the OPV (%), was moderate in its contribution. The *Barc57*^240^ allele, with a 20.3% OPV across cultivars, is not favorable for PHS tolerance. *Barc57*^220/240^ and *Barc57*^240^ should, therefore, be considered for positive and negative MAS, respectively.

#### Barc12

SSR marker *Barc12* amplified six different alleles across the cultivars studied (Table 3). Two favorable alleles for PHS tolerance, namely *Barc12*^240^ and *Barc12*^220^, were identified with 59.4 and 54.7% OVP contributions, and average PHS values of 2.6 and 2.9, respectively. The four other alleles (*Barc12*^200^, *Barc12*^160^, *Barc12*^180^, and *Barc12*^210^) are moderate contributing alleles with an OPV range of 32.8–40.6% and average PHS scores of 3.8, 3.9, 4.3, and 4.3, respectively. SSR *Barc12* alleles 240 and 220 should be considered more favorable for MAS to improve PHS tolerance.

Seven and four alleles, respectively, were amplified for the respective flanking 4A QTL SSR markers *Wmc650* and *DuPw004* (Table 4). No clear favorable single alleles below the 3.0 PHS threshold could be identified for any of these markers.

**Table 4:** Analysis of markers *Wmc650* and *DuPw004* that flank the 4A QTL to identify favorable alleles for PHS tolerance and the relative observed phenotypic variation (%) based on rain simulator screening of 96 wheat cultivars.

***Wmc650***				***DuPw004***			
**Allele**	Mean PHS score^*^	OPV%^**^	**Range**	**Allele**	**Mean PHS score**	**OPV%**	**Range**
220	2.6	59.4	2.3–3.2	190	3.6	43.8	1.5–7.2
200	3.2	50.0	2.3–4.3	280	3.8	40.6	2.0–6.8
235	3.5	45.3	1.5–7.2	190/280	4.5	29.7	3.6–5.4
170	3.7	40.3	1.9–5.4				
Null	4.0	37.5	2.7–5.4				
210	4.1	35.9	2.4–5.6				
260	5.6	12.5	5.4–5.8				

* *PHS, Pre-harvest sprouting*.

** *OPV, Observed phenotypic variation*.

#### Wmc650

Three alleles, namely *Wmc650*^210^, *Wmc650*^220^, *Wmc650*^235^ are more favorable than the other four alleles that amplified (Table 4). These three alleles contributed between 48.4 and 51.6% to the OPV and had PHS averages of 3.3, 3.1, and 3.2, respectively. *Wmc650*^260^ contributed 12.5% OPV and cultivars with this allele present had an average PHS score of 5.6. *Wmc650*^260^ is unfavorable for PHS tolerance. *Wmc650*^170^, *Wmc650*^200^, and *Wmc650*^null^ reacted moderately with PHS averages of 4.3, 3.9, and 3.6, respectively. MAS *Wmc650*^260^ should be avoided when breeding for cultivars with PHS tolerance.

#### DuPw004

Allele *DuPw004*^190^ is more favorable than the other three alleles of *DuPw004* with an OPV contribution of 48.4% and a PHS average of 3.3 (Table 4). Alleles *DuPw004*^280^*, DuPw004*^190/280^, and *DuPw004*^null^ contributed 34.4–37.5% to the OPV with PHS scores of 4.2, 4.2 and 4.0, respectively. These three alleles are not favorable for the improvement of PHS tolerance in South African germplasm.

### Favorable haplotype identification

For the whole haplotype analysis, the particular haplotypes were classed in the same manner than the single marker alleles based on the phenotypic PHS evaluation scale (Tolerant ≤ 3.0, Moderate 3.1–4.5 and Susceptible ≥4.6). The moderate class still remains difficult to define with a relevant score threshold as environmental effects might have a bigger influence on this group of cultivars than the other two classes. Cultivars from the moderate class can, depending on season and environment, move between classes.

#### Favorable haplotype identification for the 3A QTL

After the analyses of the allelic SSR marker data across the 3A QTL region, a total of 13 different haplotypes were observed (Table 5). Haplotypes 1 and 2 are considered favorable for PHS tolerance, both with PHS averages of 3.0 and an OPV (%) range of 53.1, respectively. The moderately favorable alleles from single SSR allele analysis, namely *Barc57*^220^, *Barc12*^220^, and *Barc12*^160^, are the contributors to favorable haplotypes 1 and 2. These marker alleles contribute additively to haplotypes 1 and 2 with overall improvements in the PHS averages.

**Table 5:** Analyses of the haplotype combinations for the 3A QTL across markers *Barc57* and *Barc12* to determine favorable haplotypes for PHS tolerance and the relative observed phenotypic variation (%) based on rain simulator screening of 96 wheat cultivars.

**Haplotype**	***Barc57***	***Barc12***	Mean PHS score^*^	OPV%^**^
1	220	220	3.0	53.1
2	220	160	3.0	53.1
3	220	210	3.2	50.0
4	210	200	3.4	46.9
5	220	180	3.4	46.9
6	210/240	200	3.4	43.8
7	220/240	220	3.5	45.3
8	210	160	3.8	40.6
9	220/240	200	3.8	40.6
10	210/240	210	4.3	39.1
11	220/240	240	4.3	39.1
12	220/240	160	4.8	37.5
13	220/240	180	4.8	34.4

* *PHS, Pre-harvest sprouting*.

** *OPV, Observed phenotypic variation*.

Haplotypes 3 to 11 are considered moderate contributing haplotypes toward PHS tolerance in South African cultivars for the 3A QTL region (Table 5). Haplotype 3 is a favorable moderate haplotype with a PHS average of 3.2, consisting of two moderate marker alleles *Barc57*^220^ and less favorable moderate marker allele *Barc12*^210^, suggesting additive allele interactions. Haplotype 4 is a combination of the moderate *Barc57*^210^ allele with the moderate *Barc12*^200^ allele, while haplotype 5 is a combination of the more favorable moderate *Barc57*^220^ allele and the less favorable *Barc12*^180^ moderate allele. Haplotype 6 is a combination of the less favorable moderate *Barc57*^210/240^ and the more favorable moderate *Barc12*^200^ allele combination. Haplotypes 4, 5, and 6 had average PHS scores of 3.4 with 46.9% OVP (%). Haplotype 5, with PHS score of 3.5 and OVP (%) of 45.3% is comprised of two moderate alleles, namely the less favorable *Barc57*^220/240^ and the more favorable *Barc12*^220^ allele. Haplotypes 8 to 11 are classed as less favorable moderate haplotypes with PHS averages of 3.8, 3.8, 4.3, and 4.3, respectively. These four haplotypes are all different combinations of less favorable moderate alleles from both flanking markers.

Haplotypes 12 and 13 are susceptible haplotypes both with 4.8 PHS averages. These two haplotypes are made up of the less favorable moderate marker allele combinations *Barc57*^220/240^ and *Barc12*^160^ and *Barc12*^180^. These SSR allele combinations of haplotypes 12 and 13 appear to have negative interactions or contribute susceptibility factors as the PHS score averages are higher (indicating more susceptibility) than the single moderate contributing SSR marker alleles.

For the 3A QTL region, haplotypes 1, 2, and 3 can be considered for potential MAS to improve PHS tolerance. Haplotypes 12 and 13 can be targeted negatively in MAS and should strictly be avoided during germplasm development.

#### Favorable haplotype identification for the 4A QTL

Analyses of the allelic SSR marker data across the 4A QTL region, identified ten different haplotypes (Table 6). Haplotypes 1 and 2 are considered highly favorable tolerant haplotypes for PHS tolerance with PHS average scores of 2.2 and 2.6, respectively. Haplotype 1 is a unique combination of two strong moderate alleles *Wmc650*^170^ and *DuPw004*^190^, working additively to confer a tolerant haplotype. The change of two moderately favorable marker alleles to a favorable haplotype elucidates to strong additive effects in this 4A QTL region or across both QTL regions. Haplotype 2 is a combination of tolerant marker allele *Wmc650*^220^ and moderate allele *DuPw004*^190^ with negating contributing effects to the haplotype PHS average. Allele *Wmc650*^220^ (Table 4) shows a dominant effect on haplotype 2 with a mean PHS score of 2.6.

**Table 6:** Analyses of the haplotype combinations for the 4A QTL across markers *Wmc650* and *DuPw004* to determine favorable haplotypes for PHS tolerance and the relative observed phenotypic variation (%) based on rain simulator screening of 96 wheat cultivars.

**Haplotype**	***Wmc650***	***DuPw004***	Mean PHS score^*^	OPV%^**^
1	170	190	2.2	65.6
2	220	190	2.6	59.4
3	200	190	3.2	50.0
4	235	190	3.5	45.3
5	170	280	3.7	42.2
6	Null	280	3.8	40.6
7	210	190	4.1	35.9
8	Null	190	4.3	32.8
9	170	190/280	4.5	29.7
10	260	190	5.6	12.5

* *PHS, Pre-harvest sprouting*.

** *OPV, Observed phenotypic variation*.

Haplotypes 3 and 4, with PHS mean values of 3.2 and 3.5, respectively (Table 6), are less favorable than haplotypes 1 and 2 for the 4A QTL region and as a result are classified as moderate haplotypes. Both these haplotypes are combinations of moderate contributing alleles for both markers *Wmc650* and *DuPw004*. Haplotypes 5 (PHS = 3.7), 6 (PHS = 3.8), 7 (PHS = 4.1), and 8 (PHS = 4.3) are less favorable moderate haplotypes. These four haplotypes consist of combinations of less favorable moderate marker alleles and contribute less favorably to PHS tolerance than haplotypes 1, 2, 3, or 4.

Haplotypes 9 and 10 are unfavorable for PHS tolerance with mean PHS scores of 4.5 and 5.6, respectively. Haplotype 9 consists of two strong moderate alleles namely *Wmc650*^170^ and *DuPw004*^190/280^ (Table 4), while haplotype 10 contains the moderately favorable *DuPw004*^190^ allele and the susceptible *Wmc650*^260^ allele.

Haplotypes 1, 2, and 3 should be considered for potential use in MAS for PHS tolerance, while haplotypes 4, 5, 6, 7, and 8 should be avoided if possible to eliminate the potential moderate PHS class as the moderate class tends to be strongly influenced by environmental factors. Haplotypes 9 and 10 can be targeted negatively for MAS when trying to improve PHS tolerance in new germplasm.

#### Additive haplotype combination identification across 3A and 4A QTL

When haplotype combinations for both the 3A and 4A QTL regions combined were considered, 13 different haplotypes were observed after analyses (Table 7). The majority of the cultivars (58%) were classed into haplotype combinations 1, 2, 5, 7, and 8. Two clear favorable additive (tolerant) haplotypes for PHS tolerance, namely haplotypes 1 and 2 both with PHS average scores of 2.7 and OVP (%) contributions of 57.8%, were identified. Haplotype 1 (Table 7) is comprised of the favorable 3A QTL haplotype 1 (Table 5) and the moderately favorable 4A QTL haplotype 4 (Table 6). Haplotype 2 (Table 7) is an additive combination of the 3A QTL haplotype 2 (Table 5) and 4A QTL haplotype 5 (Table 6).

**Table 7:** Analyses across both 3A and 4A QTL to identify additive haplotype combinations for PHS tolerance and the relative observed phenotypic variation (%) based on rain simulator screening of 96 wheat cultivars.

**Haplotype combination**	**Number of cultivars**	***Barc57***	***Barc12***	***Wmc650***	***DuPw004***	Mean PHS score^*^	OPV%^**^
1	8	220	220	235	190	2.7	57.8
2	7	220	160	170	280	2.7	57.8
3	3	210/240	200	170	280	3.1	51.6
4	4	220	180	235	190	3.4	46.9
5	8	220/240	220	235	190	3.5	45.3
6	2	210	200	Null	280	3.7	42.2
7	6	210	160	210	190	3.8	40.6
8	8	220/240	200	170	280	3.8	40.6
9	3	220/240	220	Null	190	4.0	37.5
10	2	220/240	200	Null	280	4.0	37.5
11	5	220/240	240	235	190	4.0	37.5
12	5	210/240	210	170	280	4.3	29.7
13	3	220/240	160	260	190	5.6	12.5

* *PHS, Pre-harvest sprouting*.

** *OPV, Observed phenotypic variation*.

Haplotypes 3, 4, and 5 are moderately favorable for PHS tolerance with PHS averages of 3.1, 3.4, and 3.5, respectively. These three haplotypes consist of different combinations of favorable and moderately favorable haplotypes. Haplotypes 3, 4 and 5 with OPV (%) in the range of 45.3–51.6% still contributed significantly to the observed phenotypic variation for PHS tolerance. Haplotypes 3, 4, and 5 (Table 7) consist of different combinations of moderate haplotypes from 3A and 4A QTL.

Haplotypes 6, 7, and 8 (Table 7) are shown to be less favorable moderate haplotypes across both the 3A and 4A QTL regions, with average PHS scores of 3.7, 3.8, and 3.8, respectively. Haplotypes 9, 10, 11, and 12 are strong moderate haplotypes with average PHS scores of 4.0, 4.0, 4.0, and 4.3, which are less favorable for PHS tolerance. The OPV (%) contribution range of 29.7–37.5%, resulted from different combinations of moderate haplotypes from both the 3A QTL and 4A QTL.

It is important to note that the susceptible haplotype 13 (Table 5) of the 3A QTL region and the strong moderate haplotype 9 (Table 6) for the 4A QTL region, did not appear regularly in any haplotype combinations across the 3A and 4A QTL region (Table 7).

The highly unfavorable susceptible haplotype 13 (Table 7) with an average PHS value of 5.6 contributed a low 12.5% toward the PHS tolerance observed. It is comprised of the susceptible haplotype combination of haplotype 12 (Table 5) for the 3A QTL and haplotype 10 (Table 6) for 4A QTL region.

### PHS class prediction based on SSR marker data

Only haplotype combinations that were present in two or more of the cultivars were considered for analysis. Haplotypes were considered unique when different combinations of the representative haplotypes in 3A and 4A QTL analysis only appeared once, or when a totally unique single SSR marker allele was present in the genotype. The results of the PHS prediction based on marker haplotypes are shown in Table 8A for dryland cultivars and Table 8B for irrigation cultivars. In these tables the cultivars with unique haplotypes were removed and were not used in the prediction. In the end, 64 cultivars of the original 96 were used in the prediction of PHS.

**Table 8A:** PHS tolerance class prediction based on molecular marker haplotype combinations across 3A and 4A QTL on the dryland cultivars used in this study.

**Dryland cultivar**	**Haplotype combination**	PHS^*^ score prediction	**Predicted PHS class**	**Actual mean PHS score**	**Actual PHS class**
Betta	1	2.7	Tolerant	1.5	Tolerant
Betta-DN	1	2.7	Tolerant	2.1	Tolerant
Elands	2	2.7	Tolerant	2.0	Tolerant
Gariep	5	3.5	Moderate	3.5	Moderate
Karee	2	2.7	Tolerant	2.1	Tolerant
Komati	5	3.1	Moderate	2.0	Tolerant
Koonap	2	2.7	Tolerant	3.9	Moderate
Letaba	7	3.8	Moderate	3.2	Moderate
Limpopo	3	3.1	Moderate	3.1	Moderate
Matlabas	2	2.7	Tolerant	2.7	Tolerant
Molopo	2	2.7	Tolerant	3.2	Moderate
PAN 3111	9	4.0	Moderate	4.4	Moderate
PAN 3118	7	3.8	Moderate	3.8	Moderate
PAN 3122	8	3.8	Moderate	4.5	Moderate
PAN 3144	1	2.7	Tolerant	2.7	Tolerant
PAN 3161	6	3.7	Moderate	4.5	Moderate
PAN 3198	8	3.8	Moderate	4.5	Moderate
PAN 3355	2	2.7	Tolerant	3.0	Tolerant
PAN 3377	4	3.4	Moderate	3.3	Moderate
PAN 3379	1	2.7	Tolerant	3.6	Moderate
Senqu	1	2.7	Tolerant	2.7	Tolerant
SST 316	4	3.4	Moderate	3.8	Moderate
SST 356	4	3.4	Moderate	3.5	Moderate
SST 374	2	2.7	Tolerant	3.0	Tolerant
SST 387	7	3.3	Moderate	3.8	Moderate
SST 398	9	4.0	Moderate	2.7	Tolerant
SST 399	6	3.7	Moderate	2.8	Tolerant
SST 936	1	2.7	Tolerant	3.5	Tolerant
Tugela	11	4.0	Moderate	7.2	Susceptible
Tugela-DN	11	4.0	Moderate	6.4	Susceptible

* *PHS, Pre-harvest sprouting*.

**Table 8B:** PHS tolerance class prediction based on molecular marker haplotype combinations across 3A and 4A QTL on the irrigation cultivars used in this study.

**Irrigation cultivar**	**Haplotype combination**	PHS^*^ score prediction	**Predicted PHS class**	**Actual mean PHS score**	**Actual PHS class**
Adam Tas	13	5.6	Susceptible	5.8	Susceptible
Biedou	8	3.8	Moderate	2.9	Tolerant
Chokka	12	4.3	Moderate	4.6	Susceptible
CRN 826	12	4.3	Moderate	4.4	Moderate
Duzi	5	3.5	Moderate	3.7	Moderate
Gamtoos	10	4.0	Moderate	3.9	Moderate
Inia	10	4.0	Moderate	4.1	Moderate
Kariega	11	4.0	Moderate	2.5	Tolerant
Marico	7	3.8	Moderate	3.1	Moderate
Nantes	8	3.8	Moderate	3.9	Moderate
Olifants	7	3.8	Moderate	4.9	Susceptible
Palmiet	12	4.3	Moderate	4.4	Moderate
PAN 3434	5	3.5	Moderate	3.5	Moderate
PAN 3471	9	4.0	Moderate	4.9	Susceptible
PAN 3478	5	3.5	Moderate	3.3	Moderate
PAN 3489	8	3.8	Moderate	4.8	Susceptible
PAN 3497	5	3.5	Moderate	3.4	Moderate
PAN 3515	8	3.8	Moderate	3.2	Moderate
Sabie	1	2.7	Tolerant	2.8	Tolerant
SST 38	3	3.1	Moderate	2.9	Tolerant
SST 806	11	4.0	Moderate	4.8	Susceptible
SST 822	12	4.3	Moderate	3.8	Moderate
SST 825	13	5.6	Susceptible	5.4	Susceptible
SST 866	8	3.8	Moderate	4.0	Moderate
SST 867	7	3.8	Moderate	2.5	Tolerant
SST 876	13	5.6	Susceptible	5.6	Susceptible
SST 877	4	3.4	Moderate	2.3	Tolerant
SST 884	11	4.0	Moderate	4.7	Susceptible
SST 33	12	4.3	Moderate	4.5	Moderate
SST 86	3	3.1	Moderate	3.3	Moderate
T4	1	2.7	Tolerant	2.3	Tolerant
Tamboti	8	3.8	Moderate	3.4	Moderate
Timbavati	5	3.5	Moderate	3.3	Moderate
Umlazi	5	3.5	Moderate	3.3	Moderate

* *PHS, Pre-harvest sprouting*.

#### Dryland cultivar predictions

Thirty of the 47 dryland cultivars could be assigned to a specific haplotype combination (Table 8A). Seventeen cultivars had unique haplotypes and were removed from the analyses. Of the 30 cultivars that were haplotyped, only seven did not predict the correct PHS class. In 76.7% of the time, the haplotype combinations were able to predict the correct PHS class overall for the dryland cultivars. The 30 cultivars that were haplotyped, could be divided into true PHS classes, where 13 cultivars were tolerant, 15 were moderate and two cultivars were susceptible. Within the tolerant class, 10 out of the 13 cultivars (76.9%) were predicted correctly. Within the moderate class, 12 of the 15 cultivars (80.0%) were predicted correctly. Both susceptible cultivars were incorrectly predicted as moderate.

#### Irrigation cultivar predictions

Thirty-four of the 49 irrigation cultivars could be assigned to a haplotype combination (Table 8B). Fifteen cultivars have unique or unassignable haplotype combinations based on the SSR data across both the 3A and 4A QTL and were removed. The haplotype analysis on irrigation cultivars was able to predict the correct PHS class of 67.6% of the irrigation cultivars after comparison with the actual PHS average scores. Of the 34 cultivars haplotyped, seven were classed as tolerant, 18 as moderate and nine as susceptible based on the actual PHS score averages. Two of the seven tolerant cultivars (28.6%) and three of the nine (33.3%) susceptible cultivars were predicted correctly. Of the moderate classed cultivars all 18 (100%) were predicted correctly based on the relative haplotype combination analysis. This mixture of prediction accuracy could be a result of the different environmental conditions and more complex gene interactions at play under irrigation production.

### *TaPHS1* SNP genotyping

The two diagnostic causal SNP mutation markers of the *TaPHS1* gene region, *TaPHS1-646* and *TaPHS1-666*, were screened on the 64 cultivars, which were successfully assigned a SSR haplotype combination across the 3A and 4A QTL regions (Tables 9A,B). Thirty-two cultivars were not considered for SNP genotyping based on the unique SSR haplotype combinations observed in those cultivars. With the nature of the SNP data only being able to reliably distinguish between tolerant and susceptible classes, an adjustment in prediction methodology was needed. For this SNP data analyses an actual PHS average score of 3.5 was considered a threshold between tolerant and susceptible classes. For the purpose of these analyses, a cultivar was considered tolerant with a PHS value ≤ 3.4 and susceptible with a PHS value ≥3.5.

**Table 9A:** PHS tolerance class prediction based on KASP SNP marker analyses on the dryland cultivars used in this study.

**Dryland cultivar**	**KASP Marker**		**Prediction according to marker analyses**	Mean PHS^*^ Score	Actual PHS class^**^
	***TaPHS1-646***	***TaPHS1-666***			
Betta	G	A	Tolerant	1.5	Tolerant
Betta-DN	G	A	Tolerant	2.1	Tolerant
Elands	G	A	Tolerant	2.0	Tolerant
Gariep	–	A	Unknown	3.5	Susceptible
Karee	G	A	Tolerant	2.1	Tolerant
Komati	G	A	Tolerant	2.0	Tolerant
Koonap	A	A	Susceptible	3.9	Susceptible
Letaba	A/G	T	Susceptible	3.5	Susceptible
Limpopo	G	A	Tolerant	3.1	Tolerant
Matlabas	G	A	Tolerant	2.7	Tolerant
Molopo	G	A	Tolerant	3.2	Tolerant
PAN 3111	–	–	Unknown	4.4	Susceptible
PAN 3118	A	T	Susceptible	3.8	Susceptible
PAN 3122	A/G	A	Susceptible	4.5	Susceptible
PAN 3144	A	A	Susceptible	2.7	Tolerant
PAN 3161	–	T	Susceptible	4.5	Susceptible
PAN 3198	A	T	Susceptible	4.5	Susceptible
PAN 3355	G	A	Tolerant	3.0	Tolerant
PAN 3377	G/A	A	Susceptible	3.3	Tolerant
PAN 3379	A	T	Susceptible	3.6	Susceptible
Senqu	G	A	Tolerant	2.7	Tolerant
SST 316	G	T	Susceptible	3.8	Susceptible
SST 356	G	T	Susceptible	3.5	Susceptible
SST 374	A/G	T	Susceptible	3.0	Tolerant
SST 387	A	A	Susceptible	3.8	Susceptible
SST 398	G	A	Tolerant	2.7	Tolerant
SST 399	G	A	Tolerant	2.8	Tolerant
SST 936	A/G	T	Susceptible	3.5	Tolerant
Tugela	A	T	Susceptible	7.2	Susceptible
Tugela-DN	A	T	Susceptible	6.4	Susceptible

* *PHS, Pre-harvest sprouting*.

** *Class category at a cut-off value of 3.5*.

**Table 9B:** PHS tolerance class prediction based on KASP SNP marker analyses on the irrigation cultivars used in this study.

**Dryland cultivar**	**KASP Marker**		**Prediction according to marker analyses**	Mean PHS^*^ score	Actual PHS class^**^
	***TaPHS1-646***	***TaPHS1-666***			
Adam Tas	G/A	A	Susceptible	5.8	Susceptible
Biedou	G	A	Tolerant	2.9	Tolerant
Chokka	G	A	Tolerant	4.6	Susceptible
CRN 826	G	T	Susceptible	4.4	Susceptible
Duzi	G	A	Tolerant	3.7	Susceptible
Gamtoos	G	T	Susceptible	3.9	Susceptible
Inia	A	A	Susceptible	4.1	Susceptible
Kariega	G	A	Tolerant	2.5	Tolerant
Marico	A	A	Susceptible	3.1	Tolerant
Nantes	G	T	Susceptible	3.9	Susceptible
Olifants	A	T	Susceptible	4.9	Susceptible
Palmiet	G	A/T	Susceptible	4.4	Susceptible
PAN 3434	G	T	Susceptible	3.5	Susceptible
PAN 3471	A	T	Susceptible	4.9	Susceptible
PAN 3478	A/G	T	Susceptible	3.3	Tolerant
PAN 3489	A/G	T	Susceptible	4.8	Susceptible
PAN 3497	A/G	T	Susceptible	3.4	Tolerant
PAN 3515	G	A	Tolerant	3.2	Tolerant
Sabie	G	A	Tolerant	2.8	Tolerant
SST 38	G	A	Tolerant	2.9	Tolerant
SST 806	–	T	Susceptible	4.8	Susceptible
SST 822	A	T	Susceptible	3.8	Susceptible
SST 825	A	T	Susceptible	5.4	Susceptible
SST 866	A	T	Susceptible	4.0	Susceptible
SST 867	G	A	Tolerant	2.5	Tolerant
SST 876	A	A	Susceptible	5.6	Susceptible
SST 877	A	A	Susceptible	2.3	Tolerant
SST 884	A	T	Susceptible	4.7	Susceptible
SST33	G	T	Susceptible	4.5	Susceptible
SST86	G	A	Tolerant	3.3	Tolerant
T4	G	A	Tolerant	2.3	Tolerant
Tamboti	A	A	Susceptible	3.4	Tolerant
Timbavati	G	A	Tolerant	3.3	Tolerant
Umlazi	–	A	Unknown	3.3	Tolerant

* *PHS, Pre-harvest sprouting*.

** *Class category at a cut-off value of 3.5*.

PHS susceptibility is based on the presence of one or both of the unfavorable alleles A (for the *TaPHS1-646* marker) and T (for the *TaPHS1-666* marker).

The international tolerant sources AC Domain, RL4137, Rio Blanco and Renan all amplified the favorable SNP alleles for PHS tolerance, namely the G allele for *TaPHS1-646* and the A allele for *TaPHS1-666*. The tolerant source, Transvaal, had a mixed haplotype with the favorable SNP allele at *Ta-PHS1-646*, but is heterozygous with a T/A SNP allele at the *TaPHS1-666*. The local tolerant cultivar, Elands, contained both favorable SNP alleles for PHS tolerance. Tugela-DN, which is the local susceptible check, contained the complete susceptible haplotype across the *TaPHS1* gene region, with the A allele and T allele present for *TaPHS1-646* and *TaPHS1-666*, respectively.

#### Dryland cultivar predictions based on TaPHS1 SNP genotyping

Thirty dryland cultivars were screened with both SNP markers *TaPHS1-646* and *TaPHS1-666* (Table 9A). Some cultivars gave reliability difficulties on each of the markers. After several reaction and procedural repeats, two cultivars (Gariep and PAN 3111) still had missing data and were referred to as unknown in terms of a prediction as explained in section KASPR Marker Genotyping. According to this methodology, the cultivar PAN 3161 was predicted as susceptible based on the presence of one unfavorable allele.

From the joint SNP data of markers *TaPHS1-646* and *TaPHS1-666*, 24 of the 28 (85.7%) cultivars were predicted into the correct PHS classes based on this analysis. This equates to a 9% improvement in prediction accuracy from the SSR haplotype predictions on the same dryland cultivars (Table 8A). Twelve of the 16 tolerant dryland cultivars were accurately predicted (75.0%) and all 12 susceptible cultivars were correctly predicted as susceptible (100%).

#### Irrigation cultivar predictions based on TaPHS1 SNP genotyping

The genotypic SNP data and PHS class prediction of the 34 irrigation cultivars is presented in Table 9B. These predictions are based solely on the SNP data. The cultivar Umlazi was treated as unknown in the prediction class because of unreliable and missing SNP data as discussed in section KASPR Marker Genotyping. The cultivar SST 806 was predicted as susceptible based on the presence of the unfavorable allele for the *TaPHS1-666* marker according to the methodology explained previously.

Twenty-six of the remaining 33 cultivars (78.8%) were predicted correctly into tolerant or susceptible classes after comparison with the actual PHS scores. This is an 11% percent accuracy improvement from the 67.6% class prediction accuracy achieved with SSR haplotype data analysis. Nine of the 14 tolerant cultivars (64.3%) were predicted correctly and 17 of the 19 susceptible cultivars (89.5%) were accurately predicted as susceptible.

Across all cultivars, the *TaPHS1* SNP data predicted 70% of the tolerant cultivars and 94% of susceptible cultivars correctly, based on the 3.5 PHS average threshold.

## Discussion

The cultivars that were assessed in this study were released by three different seed companies and represented diverse genetic backgrounds and growth types. All wheat cultivars grown in South Africa are red wheat types with exceptional bread making quality characteristics (Smit et al., 2010).

The PHS tolerance levels in South African wheat cultivars has steadily improved directly or indirectly through wheat breeding over the past 25 years (Barnard et al., 2005; Smit et al., 2010). As a result of continuous evaluations and adaptations to the respective breeding programmes, the PHS tolerance of cultivars improved to such an extent that only three of the dryland cultivars that are currently commercially available, have poor PHS tolerance compared to the almost 60% of cultivars with poor PHS tolerance in 1991 (Barnard et al., 1997). It is well-known that environmental conditions during grain filling can have a large effect on the expression of dormancy (Biddulph et al., 2007). Research by Biddulph et al. (2005, 2007) has shown that drought conditions during grain filling might increase dormancy in certain cultivars (Mares and Mrva, 2014). These phenomena could explain the high variation in the PHS levels of the moderate group of cultivars, especially in the dryland production regions where sporadic periods of moisture stress and high temperatures are experienced. Opposed to the higher PHS levels in dryland cultivars, it has been shown over many years that cultivars grown under irrigated conditions in South Africa do not display the same levels of tolerance. However, in these irrigated production areas moisture stress is not a factor. Previous research by Biddulph et al. (2007) has shown that a reduction in dormancy can occur when the water supply was high during the later stages of grain filling. Therefore, the sufficient supply of water at critical growth stages might reduce dormancy, possibly explaining the lower levels of PHS observed in irrigation cultivars in South Africa.

This study was the first to investigate the distribution and effect of known major QTL for PHS tolerance in South African wheat cultivars. The well-documented 3A and 4A QTL (Kulwal et al., 2005, 2012; Mares et al., 2005; Chao et al., 2010; Cao et al., 2016) were targeted for further investigation after initial screenings with several SSR markers.

According to the data from this study, the South African PHS tolerant check, Elands, compares favorably with the international sources. Based on pedigree comparisons there are no known PHS tolerance donor to confer tolerance. Data from this study are the first indication of the underlining genetic basis of the PHS tolerance in this cultivar to be predominantly as a result of the *TaPHS1* gene and other additive QTL combinations. From SSR haplotying it appears that different alleles of the contributing genes of the *Phs1-A1* locus (4A QTL) might be different from the international PHS tolerant donors.

According to previous research the 3A *Qphs.pseru-3AS* (Kulwal et al., 2005; Liu et al., 2008, 2011) and 4A QTL regions (Flintham, 2000; Mares et al., 2005; Ogbonnaya et al., 2007; Chen et al., 2008; Zhang et al., 2008; Graybosch et al., 2013; Cabral et al., 2014) contribute significantly to the partial PHS tolerance conferred by multiple genes. Additional common and stable QTL for PHS tolerance are 2A (Mohan et al., 2009), 2B (Chao et al., 2016; Fakthongphan et al., 2016), 3B, 3D (Kulwal et al., 2004; Ogbonnaya et al., 2007; Fofana et al., 2009; Jaiswal et al., 2012), 4B (Kulwal et al., 2012; Cao et al., 2016), 5A (Groos et al., 2002), 6B and 7D (Roy et al., 1999). Similar to other genetic studies that suggest that genes linked with PHS tolerance are mostly located on chromosome 3A and 4A (Graybosch et al., 2013; Cabral et al., 2014), it became clear from the current study that the effects of the 3A and 4A QTL on the phenotypic variation of PHS of South African cultivars are most important.

The four SSR markers, *Barc57, Barc12, Wmc650*, and *DuPw004* used to haplotype the studied material, identified clear single favorable marker alleles across diverse genetic backgrounds, which can be considered for MAS. The same markers for the 3A QTL region were used during the fine mapping and cloning of candidate gene *TaPHS1* (Liu et al., 2013). These markers have also been used successfully during the positional mapping of these QTL in previous studies (Singh et al., 2012; Tyagi and Gupta, 2012; Cao et al., 2016). The allelic variation identified with the four SSR markers strongly suggests the presence of different allelic versions of candidate genes or presence of novel mutations at the 3A and 4A QTL regions. The 13 haplotypes identified for the 3A QTL, as well as the 10 haplotypes for the 4A QTL, represented cultivars from all three PHS tolerance classes (tolerant, moderate and susceptible). In other mapping studies, an explained phenotypic variation for a single allele linked to PHS tolerance ranged between 15 and 45% (Hori et al., 2010; Chang et al., 2011; Liu et al., 2011). In the current study the OPV (%) range (higher than 40%) which was calculated in nine out of the 13 haplotypes for the 3A QTL and six out of the 10 haplotypes for the 4A QTL, indicated significant contributions by favorable haplotypes toward PHS tolerance. This OPV (%) range of up to almost 60% suggests the additive effect of the contribution of candidate genes within both the 3A and 4A QTL regions. From these combined analyses, it is therefore clear that additive haplotype combinations can be targeted during MAS.

Based on SSR haplotyping, cultivars were predicted to have a certain PHS tolerance. This is the first attempt to predict PHS tolerance based on molecular data in commercially available cultivars. In the case of dryland cultivars, this methodology predicted the correct PHS class in almost 77% of the time and 68% in the irrigation cultivars. Although some of the predictions based on SSR haplotyping classed cultivars incorrectly, in these cases cultivars were always classed in the group directly following or directly prior to that specific grouping and never two groupings apart. At no stage was a susceptible cultivar wrongly classed as tolerant or a tolerant cultivar wrongly classed as susceptible. The cultivars were always wrongly grouped between moderate and tolerant or moderate and susceptible classes.

Analyses based on SNP haplotyping were different, because only two PHS classing groups (tolerant and susceptible) were considered. In this case, cultivars that were phenotypically tolerant could be predicted through SNP haplotyping as susceptible or *vice versa*. Importantly, the SNP predictions were based solely on the contributions made by the *TaPHS1* gene of the 3A QTL. The contributions of the 4A QTL, *Phs1-A1* (Barrero et al., 2015; Shorinola et al., 2016), are unknown for these predictions and warrant further investigation on this germplasm. Predictions made with SNP data were more accurate than the SSR data with an improvement of almost 10% in both dryland and irrigation cultivars. Dryland cultivars were predicted correctly in 86% of the cases, while the irrigation cultivars were correct in 78% of cases. However, markers *TaPHS1-646* and *TaPHS1-666* were not completely diagnostic as mentioned by Liu et al. (2013), possibly due to the fact that South African cultivars might have novel mutations in and around the *TaPHS1* gene.

With the SNP data it was harder to correctly predict tolerant cultivars than susceptible cultivars, possibly due to the masking effect of moderate cultivars, as well as the unknown effects of the 4A QTL and potential susceptibility factors. The accurate predictions of moderately tolerant cultivars remains a challenge. However, the data from this study has given more insight into the genetic variation within the moderate class, further emphasizing the complexity of the PHS traits influenced by the environment (Kulwal et al., 2012; Liu et al., 2013; Mares and Mrva, 2014). The high success rate of predicted values of almost 82% on average are indicative of the possible application of this methodology in future PHS screenings. It appears that these data could be a preliminary indication of a cultivar's potential PHS class. However, the lack of a universal PHS evaluation scale, as well as the theoretical cut-off PHS score between classes, might influence the prediction outcomes.

In future, the SNP markers (*TaPHS1-646* and *TaPHS1-666*) specific to the 3A QTL can be used to select for better PHS tolerance cultivars. The newly published diagnostic markers for the *Phs1-A1* locus need to be validated on this set of cultivars. Potentially the combination of using targeted MAS for *TaPHS1* (3A) and *Phs1-A1* (4A) with true diagnostic markers, may improve PHS class prediction accuracy in the future. It is envisaged that this methodology will be further fine-tuned and validated with in-season phenotyping screenings and leaf material sampling to assist with PHS tolerance classification and recommendations.

The fact that the phenotypic PHS screenings of the 96 cultivars were conducted over a 25-year period and at several wheat producing localities throughout the wheat production areas of South Africa, could also have influenced the outcome of the data. It has been reported that environmental effects play a significant role in the PHS tolerance or susceptibility of certain cultivars (Barnard, 2001; Barnard et al., 2005; Barnard and Smith, 2009).

The methodology explained in this study has the potential to be applied in a MAS approach to predict the PHS tolerance class during the development of germplasm, enabling breeders to select for and release cultivars with improved PHS tolerance.

## Author contributions

Both authors have contributed to the work and agreed to be in the author list. SS is the molecular scientist and contributed molecular data and the interpretation thereof. AB, as the plant physiologist, contributed phenological PHS data and the interpretation thereof. Both authors contributed to the writing of the manuscript and preparing it for publication.

### Conflict of interest statement

The authors declare that the research was conducted in the absence of any commercial or financial relationships that could be construed as a potential conflict of interest. The reviewer FG and handling Editor declared their shared affiliation.
